# Editorial: Unicellular organisms as an evolutionary snapshot toward multicellularity

**DOI:** 10.3389/fcell.2023.1254636

**Published:** 2023-08-17

**Authors:** Pina Marotta, Antonella Ruggiero, Gust Bilcke

**Affiliations:** ^1^ Stazione Zoologica Anton Dohrn, Naples, Italy; ^2^ Developmental Biology Unit, European Molecular Biology Laboratory, Heidelberg, Germany; ^3^ VIB Center for Plant Systems Biology, Ghent, Belgium; ^4^ Department of Plant Biotechnology and Bioinformatics, Ghent University, Ghent, Belgium

**Keywords:** unicellular organism, evolution, multicellular organism, development, differentiation, cell cooperation

The emergence of multicellular organisms has been one of the major transitions during the evolutionary history of life on Earth (Smith and Szathmáry, 1995), with multicellularity evolving several times independently in the Tree of Life (Grosberg and Strathmann, 2007; Bonner, 2009; Niklas and Newman, 2013). Multicellularity can be simply defined as the result of cellular aggregation (Schirrmeister et al., 2013) or, if more stringent criteria are taken into account, as a coordinated behavior involving cell-to-cell interconnection, communication, cooperation, and differentiation (Kaiser, 2001; Wolpert and Szathmáry, 2002). For this reason, both complex organisms such as fungi, plants and animals, and, to some extent, simple microbial systems like colonial protists and biofilm-forming bacteria are considered multicellular (Grosberg and Strathmann, 2007; Hengge, 2020).

Unveiling the circumstances that caused unicellular organisms to evolve complex multicellularity and the molecular mechanisms that supported this transition is extremely challenging, considering that the first steps occurred more than 200 million years ago for plants, and 600 million years ago for animals (Herron et al., 2009; Ros-Rocher et al., 2021). Although researchers from various disciplines have studied this question for several decades, many unanswered questions remain about why unicellular organisms formed multicellular beings so frequently during evolution, which regulatory changes supported this profound change, and how organisms overcame individual self-interest in favor of altruistic or synergistic cooperation.

Typically, two changes are considered essential for the evolution of complex multicellularity: cell-cell adhesion and intercellular communication leading to coordinated action. These traits are common in unicellular species with a facultative multicellular life stage, such as green algae, fungi, slime molds, and choanoflagellates [reviewed in Brunet and King (2017)]. Indeed, many of these species show differentiation across their life cycle that, at some level, may be interpreted as developmental, although they do not involve communication among cells as sophisticated as that of multicellular organisms or cell death. Even obligate unicellular organisms can display traits that are linked to the emergence of multicellularity, such as intercellular communication, cell-cell adhesion, and differentiation into distinct developmental stages during their (a)sexual life cycles. These characteristics have been exploited in some unicellular groups, such as the yeast *Saccharomyces cerevisiae*, to explore the early phases of multicellularity through experimental evolution (Ratcliff et al., 2012).

**FIGURE 1:**
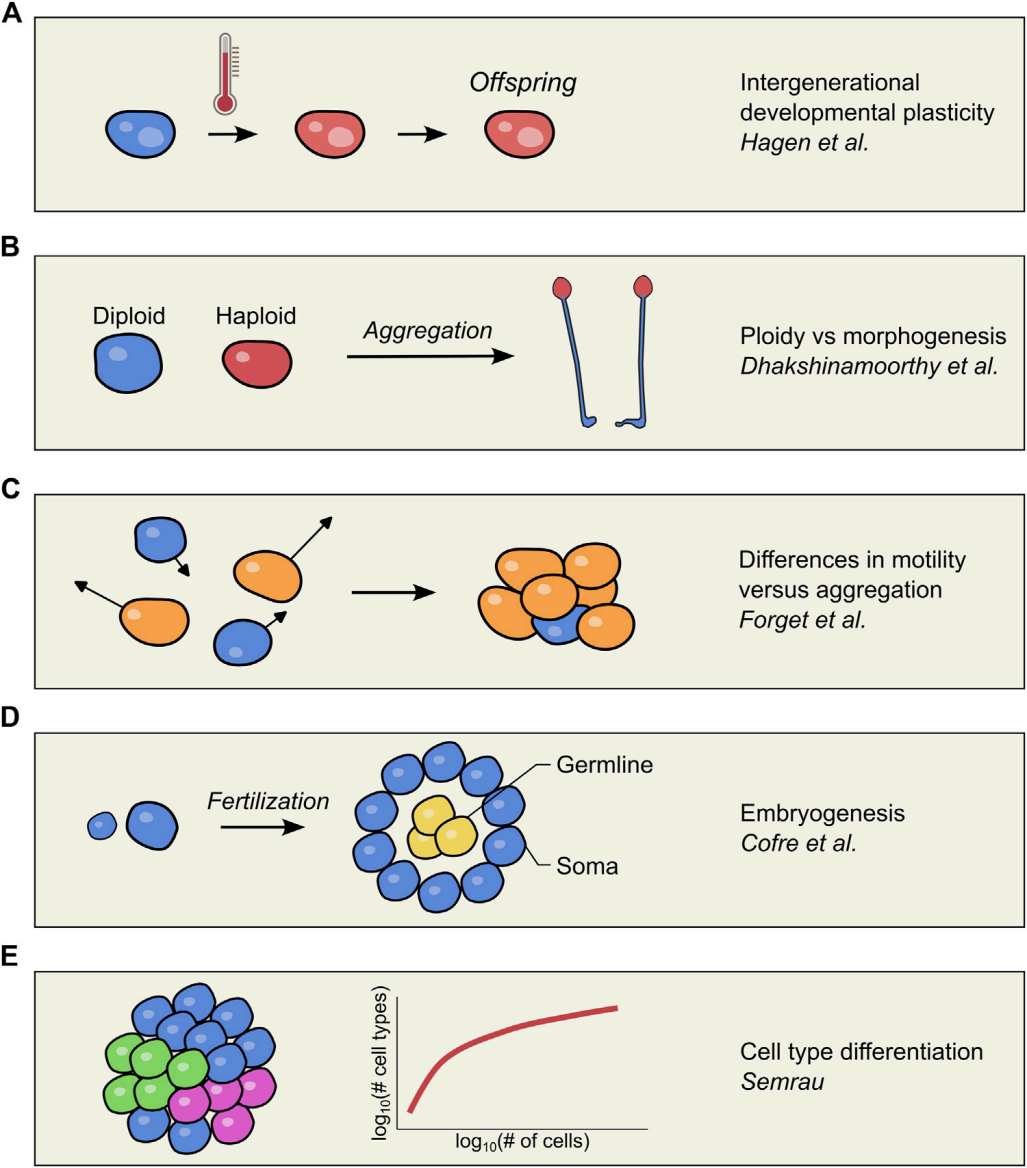
Schematic overview of the concepts investigated in each article of the Research Topic. **(A)** Somatic macronucleus development is affected by a heat shock in *Paramecium*, visualized by a red colored cell. Despite the lack of a heat shock treatment in the offspring, somatic development is still perturbed, suggesting epigenetic inheritance (Hagen et al.). **(B)** Haploid and diploid amoeboid cells were mixed to form a chimeric aggregate in the slime mold *Dictyostelium discoideum*. Cells showed a preferred localization based on their ploidy, with haploid cells differentiating into spores in the fruiting bodies (Dhakshinamoorthy and Singh). **(C)** Computer simulations explored how heterogeneity in the motility of free-living cells is reflected in the composition of the multicellular body after aggregation. The difference in motility between cells is shown through the length of arrows and the color of the corresponding cell (Forget et al.). **(D)** Cofre and Saalfeld discuss two crucial facets of embryogenesis: fertilization by unicellular gametes, and the separation of germline in yellow and the soma in blue. **(E)** The number of different cell types in a multicellular organism (colored cells) tends to scale allometrically with the number of cells in the organism (Semrau).

Through this Research Topic, “*Unicellular Organisms As An Evolutionary Snapshot Toward Multicellularity*,” which consists of three Original Research articles and two Hypothesis and Theory articles (Figure 1), we wanted to emphasize the study of unicellular and simple multicellular organisms as an additional approach to understand the molecular mechanisms at the basis of multicellularity.

Environmental conditions may affect organismal development by stimulating long-term patterns of gene expression and inducing novel phenotypes, which can be hereditary via epigenetic mechanisms (Beldade et al., 2011). In this Research Topic, Hagen et al. exploited the process of germline to soma differentiation in the single-celled ciliate *Paramecium tetraurelia* to study developmental plasticity. After sexual reproduction, a polyploid, somatic macronucleus is formed from the germline micronucleus through endoreduplication and excision of internal elements. An elegant sequence-based readout system of excision profiles during somatic development not only revealed that environmental perturbation affected somatic development, but also that it was epigenetically inherited by the F1 population. This cross-generational transfer of ecological information implies that offspring cells can tune the development of a somatic nucleus to the conditions the parent experienced. This work establishes *Paramecium* as a new model system to study the molecular basis of developmental plasticity as an alternative to multicellular and long-lived organisms.

A cell’s ploidy triggers important phenomena in nature, such as sex determination in the Hymenoptera (Heimpel and de Boer, 2008) or the division of metabolic/cellular and reproductive labors in higher metazoans (Gavrilets, 2010; Goldsby et al., 2014). Dhakshinamoorthy and Singh showed that ploidy status influences cell fate commitment in chimeric aggregates of the amoeba *Dictyostelium discoideum*, where haploid cells were more prone to differentiate into spores. Their work helped to gain new perspectives on the evolution of germ-soma distinction, using *D. discoideum* as a model to study cell type differentiation in nascent multicellular organisms.

Multicellular organisms either arise through aggregation or mitotic cell division. During aggregation, heterogeneous sparse cells rapidly and transiently group to initiate a process of cell differentiation and division of labor to survive adverse environmental conditions (Márquez-Zacarías et al., 2021). Through computational simulation models, Forget et al. investigated how heterogeneity in the motility of adhesive particles affects the outcome of cell aggregation. The study was inspired by the life cycle of the prokaryote *Myxococcus xantus* and the eukaryote *D. discoideum*, two important model species for the transition from unicellular to multicellular through aggregation. For both species, circumstances like starvation and predation induce the formation of aggregates of genetically heterogeneous cells, which assume characteristics of labor division and differentiation (Vos and Velicer, 2009; Strassmann and Queller, 2011; Forget et al., 2021). In contrast to facultative multicellular organisms which use aggregation, obligate multicellular species undergo clonal cell division to form an embryo. The origin of the first embryo is discussed in this Research Topic by Cofre and Saalfeld, which focus on two crucial processes: the fusion of unicellular gametes, and the specification of the germline. In particular, the authors argue that the fusion between unicellular organisms led to the recruitment, through topological association in the main core of the first embryo, of independently pre-existing chromosomal domains, containing basic cancer and tumor suppressor genes. As a result, the co-regulation of these contrasting cellular processes and developmental pathways synergistically induced controlled high rates of cell proliferation. Hence, embryogenesis and cancer are considered to be two sides of the same coin, and tumor suppressors had to be simultaneously recruited to keep unbridled proliferation in check.

Finally, Semrau investigated the allometric relationship between the number of cells and the number of differentiated cell types that exist in a multicellular organism. The authors suggest an exciting new model where each new cell type that evolves in an organism is associated with a fitness cost and benefit.

The articles collected in this Research Topic use simple model systems and computer simulations to investigate several aspects of the evolution of multicellularity, ranging from embryogenesis and cellular aggregation to developmental plasticity and epigenetics. Together, they illustrate the power of using simple models systems to investigate complex evolutionary questions, leading to new insights that will contribute to shedding light on the transition from the microbial world to the plant and animal kingdom, and to stimulating future research in the field of multicellularity evolution.
